# Remarks on Uterine Hemorrhage

**Published:** 1866-10

**Authors:** G. J. Townsend

**Affiliations:** South Natick


*Remarks on Uterine Hemorrhage.* By G. J. Townsend, M. D., of South Natick.

I propose to offer a few remarks upon two forms of uterine hemorrhage, to which we are especially obnoxious in our vicinity. The first form of which I would speak occurs in the unimpregnated uterus, and with nearly equal frequency in the single and the married. It is considered a hemorrhage, and not excessive menstruation, from the facts that the catamenial periods are more or less regular as to time, often remarkably so; that the menstrual flow begins moderately and normally, the patient, except in extreme cases, being able to keep about the house; that this continues, gradually diminishing, after the second or third day, until the fifth day, and that then, with remarkable regularity in many cases, the true bleeding commences. From that day, for a longer or shorter period, the sufferer is confined to the bed, and the loss of blood is often very great.

The source of this bleeding is ulceration in some form or other, or exuberant fungoid granulations, like those so common on the surface of the body in the second stage of carbuncle.

In those cases where the ulcer is on the external surface of the os and cervix, the diagnosis is easy; but in those other cases, where it is entirely within the cavity of the cervix, it is not always easy to recognize it. And ulceration, in this latter situation has given rise to the most frightful hemorrhages I have met with in the unimpregnated organ. More than once, a grave suspicion of the existence of an intra-uterine polypus has been aroused, only to be dispelled by the most positive assurance of the normal size of the uterus, the unaltered condition of the uterine cavity, and by the successful result of local treatment.

The prognosis in this form of uterine hemorrhage should, in extreme cases, always be guarded. For though it seems a simple matter enough to promote the healing of an ulcer in these regions, the constitution of the patient is often too seriously impaired before our attention is called to the disease. The usual unfavorable termination, in the cases which have come under my observation, has not been that which occurs in chlorosis and in anæmia. The great scourge of our climate, phthisis, has closed the scene in all my unfavorable cases, and the arrest of the hemorrhage has been followed by the final cessation of all catamenial flows and by the rapid development of tubercle. Therefore, when a patient shows an habitually rapid pulse, a tendency to dyspnœa, however slight, a cough, and any physical signs of tubercle, the chances of recovery are fearfully against her. If, in addition, she is not tolerant of tonics, especially iron, her case is the more desperate.

The indications for treatment are simple enough, viz. : to control the hemorrhage during its occurrence, to promote the healing of the ulceration, and to build up the general health of the patient.

The ulceration can be made to heal readily enough by the energetic or thorough application of nitrate of silver, and by the employment of sedative, alterative, and astringent applications by means of medicated pessaries and injections. When it is confined to the cavity of the cervix, the pencil of caustic requires to be introduced as far as possible, and should be sharpened by means of a wet sponge, that it may reach every part of the disease; otherwise a portion may be hidden between the rugæ of the mucous membrane, and, escaping the touch of the pencil, may render the case unnecessarily protracted and tedious. To be effectual, too, the application should be painful, the lining membrane being much more sensitive than the covering membrane of the os. It is unnecessary to enlarge upon the means of promoting the cure, as they are treated fully enough by all authorities upon the disease.

The means of arresting the hemorrhage at the period of its occurrence have not been treated of so fully, and it may not come amiss to notice a few of them.

During the first four or five days, the menstruation being usually natural, or but little in excess of nature, nothing is to be done, save to restrict the patient to a very moderate quantity of exercise. But when, upon the morning of the fourth or fifth day, the true bleeding commences, it is of vital importance to the safety of the patient to check it as soon as possible. To this end cold water injections by the rectum are of great use in two ways: by cooling down directly and constricting the uterine engorgement, and by freeing the bowel from all accumulations, which of themselves greatly aggravate the trouble.

Cold applications to the bowels and perfect rest in the recumbent posture should also by no means be neglected. But the remedy upon which I am accustomed to place my chiefest reliance is the tampon of solid alum. This should be applied well up to the os, as soon as the true hemorrhage comes on; this can easily be done by the patient, herself, and it should be allowed to remain from two to six hours, according to the severity of the symptoms. It always arrests the flow for the time, and usually for the whole present catamenial period, though if one application be not enough, there is no danger, nor usually inconvenience, in renewing it at intervals of twenty-four hours two or three times.

As to remedies by the mouth, they do not amount to much; though I have sometimes derived decided advantage from the use of alum in powder, in five-grain doses, combined with some aromatic, and repeated at short intervals, say every two hours. The stomach usually tolerates it very well.

Lead I never use, and the vegetable astringents have not much power, or else disturb the stomach, with the exception of matico, which is often a valuable tonic where the stomach bears nothing else. Iron, in the early stages of the trouble, almost invariably makes matters worse, no matter under what form it is given. Later in the case, it is often invaluable.

There is one simple remedy, which we are in the habit of considering nearly inert, from which I think I have had quite positive results, and that is the spiritus lavandulæ compositus, given in small doses, fifteen drops every three hours. It is not only an acceptable stomachic and restorative, but has a decided astringent influence, in mild cases diminishing the sanguineous discharge to a marked degree. In full doses it rarely fails to disagree, causing nausea and præcordial distress.

Stimulants, spiced wines, brandy, and the like, so often resorted to for relief from the faintness which loss of blood causes, only aggravate the disease while temporarily relieving the symptom, and are in all cases, with very rare exceptions, positively injurious and contra-indicated.

The second, and much the most immediately formidable form of uterine hemorrhage to which I would allude, is that which occurs as a consequence of delivery.

The usual story in these cases is, that the mother was overworked to the last moment, and labor begins with the whole muscular system more or less exhausted. The number of children the patient has borne has much influence in producing the trouble, the uterus wearing out, as it were, under the very frequent calls upon its energies. But some very serious cases of *post-partum* flowing have occurred, under my care, in primiparæ.

A fortnight's wash, or a week's baking for a large family are amongst the most common preliminaries of such cases. But this, again, is not an invariable rule, for one of the most insidious and dangerous cases I have ever had was that of a wealthy patient, who enjoyed an average of very good health, and who lived in every way most rationally. The constitution of the blood had probably much to do with the trouble in this case, the patient being naturally of a lax fibre, though bearing muscular exercise well enough, and accustomed to take a good deal of it.

The warning symptoms of hemorrhage are well enough known. The restless, impatient toss of the head first, the hasty ejaculation, the hemorrhagic pulse, never less than 80, often much above 100, the absence of all uterine tumor after the expulsion of the placenta, are all warnings readily recognized and sufficiently appreciated by each of us. There is, however, one symptom which has been an almost invariable attendant of my severe cases, and which I have never seen alluded to; and that is, a peculiar, indescribable odor of the placenta, membranes and lochia, very penetrating, very different from their natural odor, and that is bad enough, and like nothing else that I know of.

That this has some connection with the flowing, I am led to believe from the fact that before I recognized the frequency of the coincidence, the presence of the taint always caused a feeling of uneasiness and apprehension, too often confirmed by the subsequent course of events.

As to the means of arresting the bleeding, it is unnecessary to speak of those that are well known. Ergot, galvanism, stimulus, cold pressure on the abdomen, causing the uterus to expel its contents and to follow up its contractions afterwards, we all have used. But there are a certain proportion of desperate cases in which all these means fail us, and where it becomes a serious question what to do next. Writers tell us that the act of vomiting conduces to the safety of the patient, producing, or being followed by contraction of the uterus. Emetics have been suggested as a means of producing contraction. It has not been so in my experience, and I have grown to regard vomiting as the most formidable symptom possible. Ergot, brandy, wine, ether, hot drinks and cold, ice even, will sometimes come up as fast as we turn them down. Each act of vomiting has been followed by a relaxation of the uterus the more obstinate, by a collapse the more alarming, the uterus pouring out blood, like water from a well saturated sponge. I would here say that I always regard it of the utmost importance that the uterus should, by contractions, expel the placenta in all cases, except where there is adhesion or hour-glass contraction. With these exceptions, it is rare that we need fail in causing it to do so, and usually a few minutes' compression will cause the contraction to be permanent.

The means, then, that have never failed me in the most severe hemorrhages are, in the first place, the local application of ice. The safety of applying ice to the os uteri, in cases of non-contraction, has often been discussed. It seems to me the only element of danger in this treatment is, that it is not carried far enough. Ice, to be effectual, should be carried through the os up to the very fundus, and should be kept there; the hand and arm acting as a tampon and preventing the escape of blood from the sinuses until the uterus contracts. A cold left hand upon the abdomen materially assists in producing the contraction, and when it occurs, and not until then, be the time longer or shorter, should the uterus be allowed slowly to expel the hand and arm. Once contracted in this manner, it has never relaxed again in any of my cases.

In the second place, to allay the vomiting, to quiet the terrible restlessness, to avert the collapse, when all other means have failed, I have depended upon opium, given in small doses at very frequent intervals. Ten drops of the acetated tincture of opium, or fifteen of laudanum, with an equal quantity of the aromatic spirits of ammonia, in a very little cold water, not more than a drachm, given every fifteen minutes, constitute my usual prescription. This is very rarely rejected, and should be persevered with if it is. Two hours is the longest time I have stood over a patient waiting for the desired result; the first fifteen minutes of quiet sleep, which puts her life out of immediate danger. We can all testify as to what a load of anxiety this brief interval of repose removes from the attending physician, and the great relief it brings us.

As to the *modus operandi* of opium, I can say nothing, except perhaps in the words of Dr. Meigs, that it acts "as the great restorer of the vital forces." I certainly can add the small weight of my testimony to his when he says, alluding to these cases, "Gentlemen, be not afraid of opium."

I offer these remarks, gentlemen, not as any thing new or original, but simply as the record of my own experience upon given points, and as just so far valuable and no farther.—*Boston Medical and Surgical Journal.*

